# Mild endoplasmic reticulum stress ameliorates lipopolysaccharide-induced neuroinflammation and cognitive impairment via regulation of microglial polarization

**DOI:** 10.1186/s12974-017-1002-7

**Published:** 2017-11-28

**Authors:** Yi-wei Wang, Qin Zhou, Xiang Zhang, Qing-qing Qian, Jia-wen Xu, Peng-fei Ni, Yan-ning Qian

**Affiliations:** 1 Department of Anesthesiology, The First Affiliated Hospital of Nanjing Medical University, Nanjing, Jiangsu 210029 People’s Republic of China; 2 Department of Anesthesiology, Shanghai General Hospital, Shanghai Jiao Tong University School of Medicine, Shanghai, 200080 People’s Republic of China

**Keywords:** Endoplasmic reticulum stress, Neuroinflammation, Microglia, Neurodegeneration, Lipopolysaccharide

## Abstract

**Background:**

Neuroinflammation, which ultimately leads to neuronal loss, is considered to play a crucial role in numerous neurodegenerative diseases. The neuroinflammatory process is characterized by the activation of glial cells such as microglia. Endoplasmic reticulum (ER) stress is commonly associated with impairments in neuronal function and cognition, but its relationship and role in neurodegeneration is still controversial. Recently, it was confirmed that nonharmful levels of ER stress protected against experimental Parkinson’s disease. Here, we investigated mild ER stress-based regulation of lipopolysaccharide (LPS)-driven neuroinflammation in rats and in primary microglia.

**Methods:**

Male Sprague–Dawley (SD) rats received the intracerebroventricular injection of the ER stress activator tunicamycin (TM) with or without intraperitoneal injection of the ER stress stabilizer sodium 4-phenylbutyrate (4-PBA) 1 h before LPS administration. The levels of neuroinflammation and memory dysfunction were assessed 24 h after treatment. In addition, the effect of mild ER stress on microglia was determined in vitro.

**Results:**

Here, we found that low doses of TM led to mild ER stress without cell or organism lethality. We showed that mild ER stress preconditioning reduced microglia activation and neuronal death as well as improved LPS-induced memory impairment in rats. In addition, pre-exposure to nonlethal doses of TM in microglia showed significant protection against LPS-induced proinflammatory cytokine production and M1/2b polarization. However, sodium 4-PBA, a compound that ameliorates ER stress, ablated this protective effect in vivo and in vitro.

**Conclusions:**

Based on our findings, we conclude that the mild ER stress not only limits the accumulation of misfolded proteins but also protects tissues from harmful endotoxemia insults. Therefore, ER stress preconditioning has potential therapeutic value for the treatment of neurodegenerative diseases.

**Electronic supplementary material:**

The online version of this article (10.1186/s12974-017-1002-7) contains supplementary material, which is available to authorized users.

## Background

Uncontrolled chronic neuroinflammation is known to play a key role in the progression of diverse neurodegenerative diseases, such as Alzheimer’s disease (AD), Huntington’s disease (HD), and Parkinson’s disease (PD), by causing an over-production of proinflammatory cytokines [1, 2]. Microglia exert neurotoxic effects during neuroinflammation by establishing a feedforward inflammatory loop that ultimately leads to neurodegeneration [3, 4]. Systemic injection of lipopolysaccharide (LPS), an endotoxin isolated from bacteria, triggers neuroinflammation and amyloidogenesis in the hippocampus [5]. Thus, LPS-induced systemic inflammation in rats is frequently used as a model for studying neurodegeneration and cognitive impairment [6].

The endoplasmic reticulum (ER) is a multifunctional organelle that activates a set of signaling pathways upon encountering stress to maintain homeostasis [7, 8]. Altered ER proteostasis results in the accumulation of unfolded/misfolded proteins in the ER lumen (known as ER stress), which activates the unfolded protein response (UPR) [9]. The UPR is mediated by three main signaling branches: the protein kinase RNA-like ER kinase (PERK)–eukaryotic translation initiation factor 2α (EIF2α) axis, the inositol-requiring protein 1α (IRE1α)–spliced X-box-binding protein-1 (XBP1s) axis, and activating transcription factor (ATF)-6α axis [10, 11].

Although accumulating reports have linked the occurrence of ER stress with several neurodegenerative diseases such as PD, HD, AD, multiple sclerosis (MS), amyotrophic lateral sclerosis (ALS), and prion-related diseases (PrDs) [7, 12], whether ER stress plays a causative role in certain disease conditions is still being debated. On the basis of some contradictory findings [13, 14], a complex scenario is emerging in which activation of specific UPR signaling mechanisms may actually function as a beneficial response to reduce neurodegeneration. Several reports have shown that nonharmful levels of ER stress are protective against experimental PD [15–17]. Similarly, using an AD model, the enforced expression of the IRE1α/XBP1s pathway was shown to protect against amyloid-β toxicity to reverse memory impairment [18, 19]. In addition, the PERK signaling branch revealed a bifunctional role in a model of ALS in which EIF2α phosphorylation had a protective effect but expression of ATF4 had detrimental consequences [20, 21].

These studies suggest that in the context of neurodegenerative diseases, the contribution of the UPR is very complex and nonlinear. Some theoretical mechanisms [12, 22] have been proposed to explain these scenarios, including the idea that targeting specific UPR components may actually generate low nonlethal levels of ER stress that could actually operate as a beneficial response to maintain homeostasis. The downstream effects of the UPR has two contrasting processes—protective responses or proapoptotic programs—that depend on several parameters, including the load of misfolded proteins and subtle differences in the type, intensity, and duration of the stressors [14]. Under conditions of moderate misfolded protein accumulation, activation of the UPR operates as a beneficial reaction that reinforces protein folding, degradation, and quality control. When the buffering capacity of the UPR is inadequate to maintain ER proteostasis, the UPR shifts signals towards a terminal pathway that drives cells towards apoptosis [11, 23, 24].

This dual aspect of UPR was explained by a possible concept of ER hormesis, which involves the engagement of a preconditioning state via mild, nonlethal ER stress to induce adaptive reactions and protect the cell from a second, stronger insult [25–27]. The concept of “ER hormesis” has been applied to the fields of ischemic brain injury and neurodegeneration [15, 20, 28]. However, there has been little direct evidence of the involvement of ER hormesis in LPS-induced neuroinflammation to date. Therefore, in the present study, we investigated the effects of mild ER stress on LPS-induced neuroinflammation and cognitive impairment in rats and in primary microglia.

## Methods

### Animals

Male Sprague-Dawley rats weighing 250 g (*n* = 154) were used in this experiment. All rats were housed under specific pathogen-free conditions (ambient temperature, 22.0 ± 1.0 °C; humidity, 40%) during breeding and experiments. Food and water were available ad libitum. During all the surgical procedures, the animals were placed under general anesthesia and every effort was made to minimize the stress and number of rats. The experiments were approved by the Nanjing Medical University Animal Care and Use Committee. All experimental procedures involving animals were approved by the Institutional Animal Care and Use Committee (IACUC) of Nanjing Medical University.

### Intracerebroventricular cannula implantation

An indwelling lateral intracerebroventricular (icv) cannula for injection of pharmacological agents was implanted in the brains of rats as previously described [29, 30]. The animals were anesthetized (2.1% inspired concentration in 0.3 FiO_2_) and placed in a stereotaxic device. Guide cannulas were inserted into the lateral ventricle (coordinates of 0.8 mm posterior, 1.5 mm lateral, and 3.7 mm ventral to the bregma) and secured to the skull with dental cement [31]. Following surgery, rats were individually housed and allowed to recover in the animal facility for 14 days before experiments. Animals were handled daily to familiarize them with the investigators and to check the guide cannula.

### Behavioral tests

**Fig. 1:**
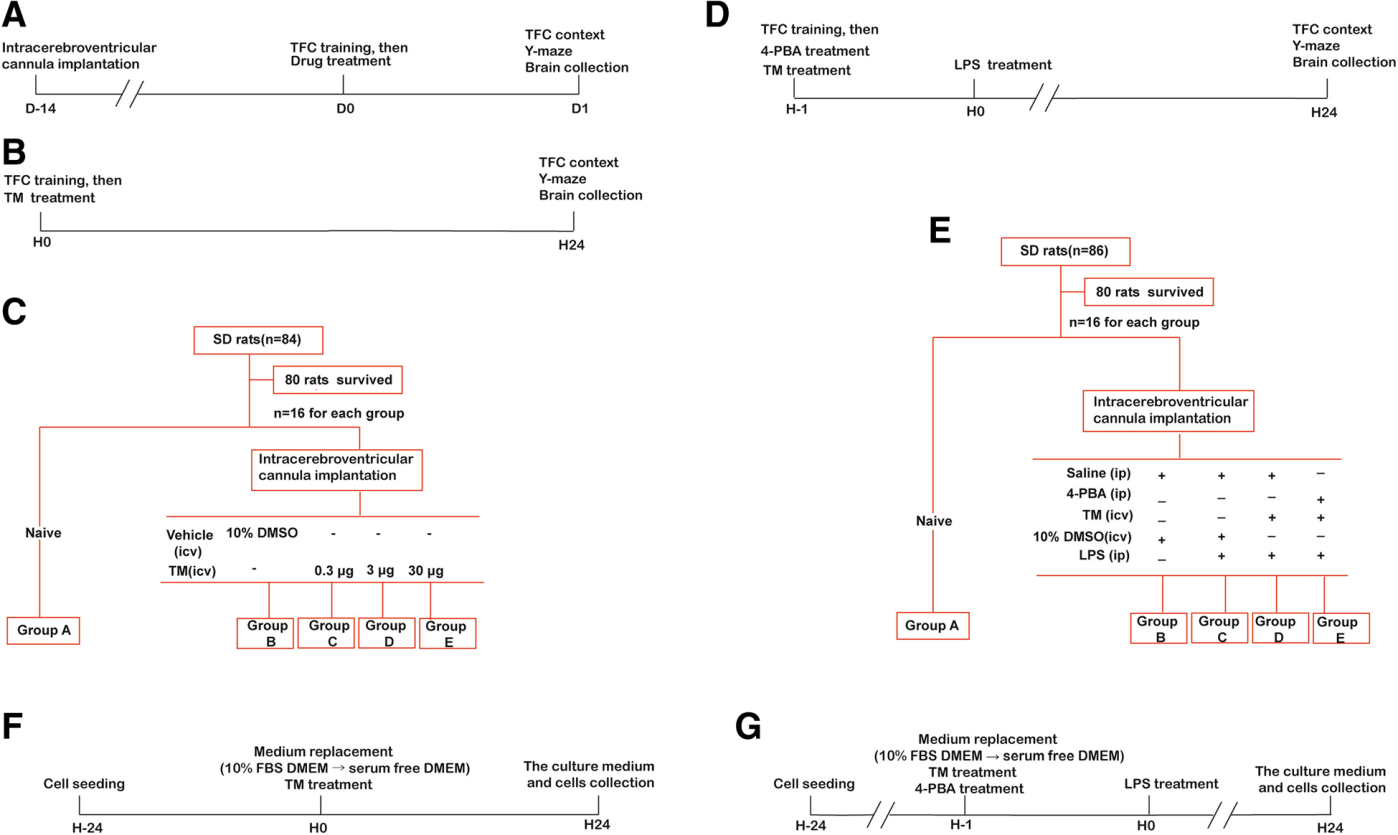
Study design. **a** Timeline of the in vivo experimental treatments. All rats underwent icv cannula implantation 14 days before use in experiments. One day after contextual fear conditioning training, all animals received drug treatments as indicated. Brains were collected 24 h after drug injection. Contextual assessment and the Y-maze test were also performed at this time point. **b**, **c** The protocol performed in the in vivo experiment 1: Groups C, D, and E received the indicated dosage of tunicamycin (TM) intracerebroventricularly immediately after contextual fear conditioning (TFC) training, while group B received an equivalent volume of vehicle. Rats of group A were naïve to all treatments. **d**, **e** The protocol performed in the in vivo experiment 2: Rats in groups C, D, and E were injected with LPS within 1 h after TFC training. Groups D and E received TM intracerebroventricularly immediately after TFC training, while groups B and C received an equivalent volume of vehicle. 4-PBA was administered by intraperitoneal (ip) injection prior to TM treatment in group E. Rats of group A were naïve to all treatments. All animals underwent behavioral testing 24 h after TM injection. Brains were collected after completion of the behavior tests. **f** The protocol performed in the in vitro experiment 1: Cells were treated with TM at the concentrations indicated for 24 h, then the culture medium and cells were collected. **g** The protocol performed in the in vitro experiment 2: Cells were pretreated with TM for 1 h followed by the addition of LPS. The culture medium and cells were collected after 24 h of incubation

The behavioral assessment lasted 2 days and consisted of three tests: trace fear conditioning (TFC), contextual assessment, and the Y-maze test. To avoid any potential influence of behavioral testing on the molecular biology experiments, molecular biology assessments and behavioral testing were performed on different animals. The behavioral test design is briefly illustrated in Fig. 1a. Complete methodological details are described in the Additional file 1: Supplemental Materials and Methods.

### Drug administration

#### Tunicamycin

TM was dissolved in 10% dimethyl sulfoxide (DMSO). The dilutions of TM (0.3, 3, and 30 μg) were prepared fresh prior to the experiment in saline containing 10% DMSO, and 2 μl of the preparation was administered via icv route [28]. This low concentration of DMSO was chosen to avoid neurological effects [32–34].

#### Sodium 4-phenylbutyrate

4-Phenylbutyric acid (4-PBA) was diluted in sterile saline and injected intraperitoneally (ip) at a dose of 100 mg/kg as previously described in the literature [35, 36].

#### Lipopolysaccharide

To induce a systemic inflammatory reaction for the experimental procedures, LPS was diluted in sterile saline and ip injected at a dose of 500 μg/kg, which elicited moderate inflammation [29]. Additionally, it has been reported that this dose does not affect motor activity [5, 37, 38].

### Experimental protocol and pharmacological treatments

The in vivo experiments consist of two parts.

#### Experiment 1

Rats were randomly assigned to one of five groups (16 rats per group), and investigators were blinded to the experimental treatment. The well-known ER stress inducer TM (0.3, 3, and 30 μg) was administered via icv cannulas in rats from groups C, D, and E immediately after TFC training. Rats in group B were administered the same volume of vehicle (10% DMSO in saline) via icv injection, while rats in group A were naïve to any treatment. The study design is briefly illustrated in Fig. 1b, c.

#### Experiment 2

The rats were randomly assigned to one of five groups (groups A–E) with 16 rats per group, and investigators were blinded to the experimental treatments. Immediately after TFC training, rats in the group E received 100 mg/kg 4-PBA intraperitoneally while the rats in groups B–D received an equivalent volume of sterile saline. Following the 4-PBA/saline treatment, a dose of 3 μg/2 μl TM (groups D and E) or 10% DMSO in saline (groups B and C) was administered via icv cannulas. After 1 h, rats were intraperitoneally challenged with sterile saline (group B) or 500 μg/kg LPS (groups C, D, and E). Rats of group A were naïve to any treatment. The study design is briefly illustrated in Fig. 1d, e.

To minimize the number of animals used in this study, experiments 1 and 2 shared the naïve rats.

The in vitro experiments consist of two parts.

#### Experiment 1

Primary microglia were seeded at 1 × 10^6^ cells in 5 cm × 5 cm flasks, and incubated for 24 h at 37 °C in a humidified atmosphere containing 5% CO_2_. The cells were treated with TM (0.5, 5, and 50 ng/ml) for 24 h, followed by collecting the culture medium and cells. The study design is briefly illustrated in Fig. 1f.

#### Experiment 2

The cells were pre-exposed to TM at 5 ng/ml with or without 4-PBA for 1 h, followed by the addition of 10 ng/ml LPS. The culture medium and cells were collected after a further 24 h of incubation. The study design is briefly illustrated in Fig. 1g.

### Primary microglial cultures and treatments

Rat microglial cultures were prepared as previously described with slight modifications [39]. Briefly, the cerebral cortex of neonatal SD rats (at postnatal day 1) was dissociated and digested with a 0.25% trypsin-EDTA solution for 10 min at 37 °C. The cells were plated in pre-coated poly-d-lysine cell culture flasks and cultured in high-glucose DMEM containing 10% fetal bovine serum (FBS) and penicillin (100 U/ml)/streptomycin (100 μg/ml). After culturing for 10 days at 37 °C in a humidified atmosphere containing 5% CO_2_ and 95% air with a medium change every 3 days, the glial cells formed a confluent monolayer. The microglial cells were separated from the astrocytes by shaking the flask for 5 h at 150 rpm to detach the layer of loosely adherent cells, which are mainly astrocytes, from the firmly adherent cells. The purity of the microglia was > 98% as confirmed by anti-Iba-1 immunochemical staining. Afterwards, the microglial cells were seeded on either poly-d-lysine pre-coated dishes or coverslips (for immunocytochemistry).

### Cell Counting Kit-8 (CCK-8) assay

Briefly, cells were seeded at a density of 5 × 10^3^ cells per well in 96-well culture plates with fresh medium containing different concentrations of reagents. Thereafter, 10 μl of CCK-8 solution was added to each well. After a 1-h incubation at 37 °C, the optical density (OD) at 450 nm was measured by a DTX-880 multimode microplate reader. Each treatment had six replicate wells, and the amount of DMSO in the reaction mixture was adjusted to be identical in each well (including the control) and never exceeded 0.1%. Moreover, each experiment was repeated at least three times.

### RT-PCR with SYBR green detection

Total RNA from the hippocampus and primary microglial cell cultures was using TRIzol reagent (Invitrogen, USA), and reverse transcription was performed as previously described. Real-time PCR amplification was performed using the STEP ONE Real-time PCR Detection System (Foster City, CA) with SYBR Green master mix (Applied Biosystems, Foster City, CA) at a final volume of 10 μl that contained 1 μl cDNA template from each sample. The PCR was carried out using the following cycling protocol: a 95 °C denaturation step for 5 min followed by 40 cycles of 95 °C denaturation (15 s), 60 °C annealing (15 s), and 60 °C extension (30 s). Detection of the fluorescent product was carried out at the end of each 60 °C extension period. The relative mRNA values were normalized to the β-actin gene control values and calculated using the comparative cycle threshold (ΔΔCt) method. The primers used are listed in Additional file 1: Supplemental Materials and Methods.

### Enzyme-linked immunosorbent assay (ELISA)

The levels of IL-1β, IL-6, and TNFα in rat hippocampal tissue extracts and the culture medium were measured using ELISA kits from R&D Systems according to the manufacturer’s instructions. Details are available in the Additional file 1: Supplemental Materials and Methods.

### Western blotting

Ipsilateral hippocampal tissues and microglial cells were homogenized in RIPA lysis buffer, which contained 20 mM Tris, 150 mM NaCl, 1% Triton X-100, 1 mM EDTA, 1.5 μg/ml leupeptin, and 1 mM phenylmethylsulfonylfluoride (PMSF). Samples were centrifuged for 20 min at 12,000×*g* (4 °C). Protein quantification was performed using the Bradford assay following the manufacturer’s guidelines (Bio-Rad). Samples were boiled for 4 min, and 20 μg of protein was separated on a 10% acrylamide gel and transferred onto nitrocellulose membranes. The membranes were blocked with 5% non-fat milk for 1 h at room temperature and incubated at 4 °C overnight with specific primary antibodies. After they were washed three times with Tris-buffered saline with Tween 20 (TBST), the membranes were incubated with secondary antibodies for 1 h at room temperature followed by three washes with TBST and then detection using a chemiluminescent substrate.

### Terminal deoxynucleotidyl transferase mediated dUTP nick end labeling (TUNEL) assay

TUNEL assay, a method for detecting DNA fragmentation, was used to measure apoptosis. The one-step TUNEL apoptosis assay kit was obtained from KeyGEN BioTECH (KGA7074). Briefly, brain sections were rinsed in 0.5% Triton X-100 in 0.01 M PBS for 20 min at 80 °C to increase permeability of the cells. To label the damaged nuclei, 50 μl of FITC-conjunctive fluorescein-12-dUTP reaction mixture was added to each sample in a humidified chamber, which was followed by a 60-min incubation at 37 °C. The nuclei were stained with DAPI. Fluorescent images were acquired using a confocal microscope.

### Immunofluorescence analysis

Upon completion of the treatment, the microglia were fixed with 4% paraformaldehyde for 30 min and blocked with 5% bovine serum albumin (BSA) in 0.1% Triton X-100 for 1 h at room temperature. Next, the cells were incubated at 4 °C with specific primary antibodies overnight. After three washes with PBS, the microglia were incubated at 37 °C with either goat anti-mouse or goat anti-rabbit Alexa Fluor 488 or Alexa Fluor 594 (as appropriate) diluted in the blocking solution for 1 h and the nuclei were stained with DAPI. The images were visualized using a confocal microscope.

Ipsilateral hippocampal tissue samples used for immunohistochemical and immunofluorescent staining were post-fixed with 4% paraformaldehyde overnight and sliced into 5-μm-thick sections using a cryostat. After they were blocked with 5% BSA, the sections were incubated with specific primary antibodies overnight at 4 °C and then incubated with PE-conjugated and/or FITC-conjugated secondary antibodies for 1 h followed by staining with DAPI. The sections were viewed under a confocal microscope.

### Immunohistochemical analysis

To determine the expression of cleaved caspase-3, ipsilateral hippocampal sections (10 μm thick) were prepared and immunohistochemistry was performed as follows. After incubation for 1 h in 10% BSA with 0.3% Triton X-100 in 0.01 M phosphate-buffered saline, the sections were incubated with specific primary antibodies overnight at 4 °C. The sections were then incubated with the appropriate secondary antibody for 2 h. Positive cells were visualized by adding DAB to the sections. Images of immunocytochemistry were digitally captured using a Leica 2500 microscope.

### Statistical analysis

The data were first tested for normality (Shapiro–Wilk test) and homoscedasticity (Levene’s test). All data were presented as either the mean ± SEM or as the median and interquartile range. Statistical analysis was performed using a one-way ANOVA followed by either a least significant difference (if the variance was equal) or Dunnett T3 (if the variance was not equal) test. *P* < 0.05 was considered statistically significant.

## Results

### Low doses of TM activated a benign, moderate ER stress response in the hippocampus of rats

TM is a commonly used ER stress inducer [28]. In this study, TM was injected intracerebroventricularly at the indicated dosage to investigate the UPR in the hippocampi.

**Fig. 2:**
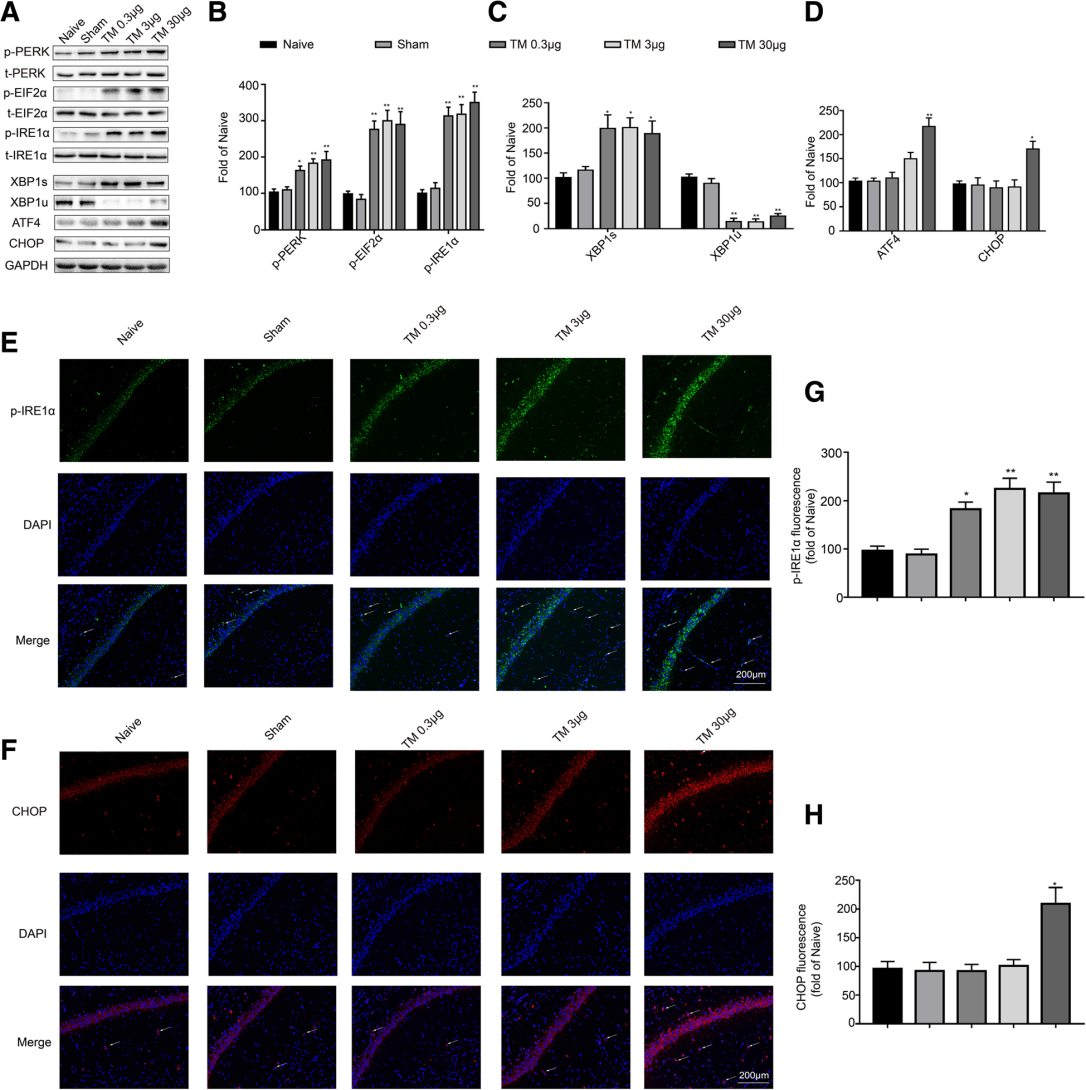
Effect of tunicamycin on the expression of UPR-related proteins in the hippocampus. **a** The expression levels of p-PERK, p-EIF2α, p-IRE1α, XBP1s, XBP1u, ATF4, and CHOP in the hippocampus of rats were detected by Western blotting using specific antibodies. Each blot is representative of three experiments. **b** Phosphorylated levels of PERK, EIF2α, and IRE1α were quantified and normalized to the corresponding total levels. **c** Expression of XBP1s and XBP1u was quantified and normalized to GAPDH expression. **d** Expression of ATF4 and CHOP was quantified and normalized to GAPDH expression. **e**, **f** Images acquired by confocal microscopy show the p-IRE1α and CHOP levels in the CA1 area of the hippocampus. The arrow in **e** points to an area in CA1 with high p-IRE1α immunoreactivity. The arrow in **f** points to an area in CA1 with high CHOP immunoreactivity. Scale bar, 200 μm. **g**, **h** Quantitative data of the mean intensities of p-IRE1α and CHOP fluorescence. Each value was expressed relative to that of the naïve group, which was set to 100 (*n* = 6). All experiments were repeated three times. *P < 0.05, **P < 0.01 vs. naïve group. The data are presented as the mean ± SEM

ER stress-activated p-IRE1α cuts unspliced XBP1 (XBP1u) mRNA into spliced XBP1 (XBP1s) mRNA, which encodes the transcriptionally active XBP1s protein [40]. To determine the activation status of IRE1 signaling after ER stress, we examined XBP1s, XBP1u, and p-IRE1α protein expression using western blotting. Significant increases in the protein expression levels of p-IRE1α and XBP1s were observed after TM (0.3, 3, and 30 μg) treatment (Fig. 2a–c). Interestingly, TM (0.3, 3, and 30 μg) treatment could reduce XBP1u expression (Fig. 2c).

PERK is activated by phosphorylation at Thr980, after which it phosphorylates EIF2α at Ser51 [10]. However, prolonged EIF2α phosphorylation induces paradoxical translation of ATF4 mRNA into the corresponding protein, which in turn induces the upregulation of pro-apoptotic components such as CHOP [40]. Thus, to further characterize the extent of PERK activation, we assessed the hippocampal expression levels of p-PERK, p-EIF2α, ATF4, and CHOP using western blotting with specific antibodies. TM (0.3, 3, and 30 μg) significantly increased the expression levels of p-PERK and p-eIF2a (Fig. 2a, b), whereas ATF4 and CHOP levels were only elevated at the highest dose of TM (30 μg). We observed that following TM icv injection of either 0.3 or 3 μg, the expression levels of ATF4 and CHOP in the rat hippocampus did not show significant changes (Fig. 2a, d).

We also examined the expression of p-IRE1α and CHOP in hippocampal CA1 using immunohistochemistry (Fig. 2e, f). Consistent with the western blotting data, increases of p-IRE1α were detected following injection of TM (0.3, 3, and 30 μg) in the hippocampal CA1 region (Fig. 2g), while administration with either 0.3 or 3 μg of TM did not affect the expression of CHOP in the hippocampal CA1 region. The CHOP levels were only elevated in response to the highest dose of TM (30 μg) (Fig. 2h). These findings showed that low doses of TM activated a benign and mild ER stress response in the hippocampus.

**Fig. 3:**
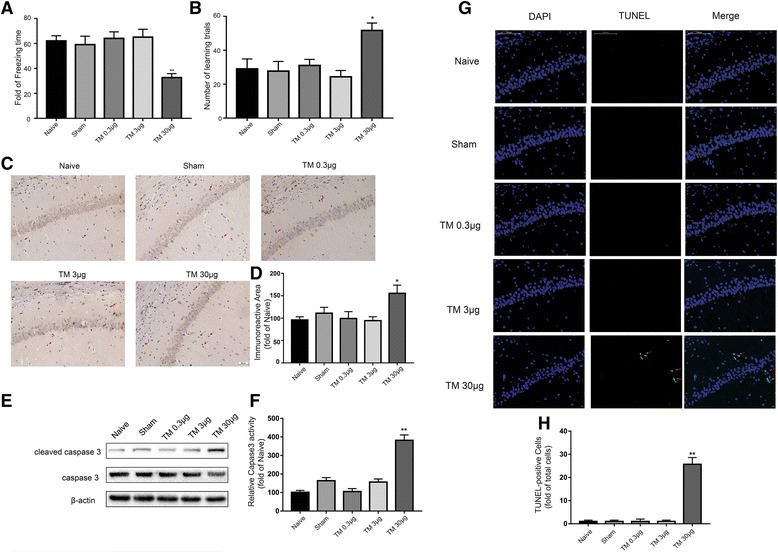
Low doses of TM activated a nonharmful, moderate UPR in the hippocampus. The freezing time in the trace fear conditioning test (**a**) and the number of learning trials in the Y-maze test (**b**) were recorded to analyze cognitive changes (*n* = 12). **c** Immunostaining was used to detect cleaved caspase-3 in the CA1 area of the hippocampus. Scale bar, 50 μm. **d** Quantification of cleaved caspase-3-positive cells in the CA1 area of the hippocampus. **e** The expression levels of cleaved caspase 3 and full-length caspase 3 in the hippocampus of rats were detected by Western blotting using specific antibodies. Each blot is representative of three experiments. **f** Quantification of cleaved caspase-3-positive cells in the CA1 area of the hippocampus. Each value was expressed relative to that in the naïve group, which was set to 100 (*n* = 6). **g** The TUNEL assay was performed to determine the extent of apoptosis in the CA1 area of the hippocampus. The arrows indicate cells showing an overlay of TUNEL and DAPI signals. Scale bar, 100 μm. **h** Quantitative analysis of TUNEL-positive cell content in different groups. *P < 0.05, **P < 0.01 vs. naïve group. The data are presented as the mean ± SEM. All experiments were repeated three times

To verify these findings, we performed contextual assessment and the Y-maze test to observe the cognitive function of the rats [29, 30]. As shown in Fig. 3a, b, rats exposed to 30 μg of TM exhibited a significant reduction in cognitive function compared to the naïve animals. However, TM injection at 0.3 and 3 μg did not change the freezing time and number of learning trials.

We tested cleaved caspase-3 using western blotting and immunostaining in the rat hippocampus. Our data indicated that only a 30-μg dose of tunicamycin increased the expression levels of cleaved caspase-3 (Fig. 3c–f).

TUNEL labeling was also performed to assess the level of apoptosis in the hippocampus. In the present study, TUNEL-positive cells were found in the 30 μg TM treatment group, but not the sham and low-dose TM (0.3 and 3 μg) groups, in the hippocampus (Fig. 3g, h). These results confirmed that low doses of TM (0.3 and 3 μg) caused a benign and moderate ER stress response in the hippocampus.

### Mild ER stress alleviated LPS-induced and neuronal apoptosis in the hippocampus and ameliorated cognitive decline

We observed that low doses of TM (0.3 and 3 μg) cause a mild UPR in the rat brain but do not induce drastic behavioral alterations or death. In the following experiment, TM was injected icv at a dose of 3 μg prior to LPS administration to induce mild ER stress in the rats. Next, we studied the effects of mild ER stress in LPS-induced neuroinflammation and cognitive impairment.

#### TM improve LPS-induced memory impairment

**Fig. 4:**
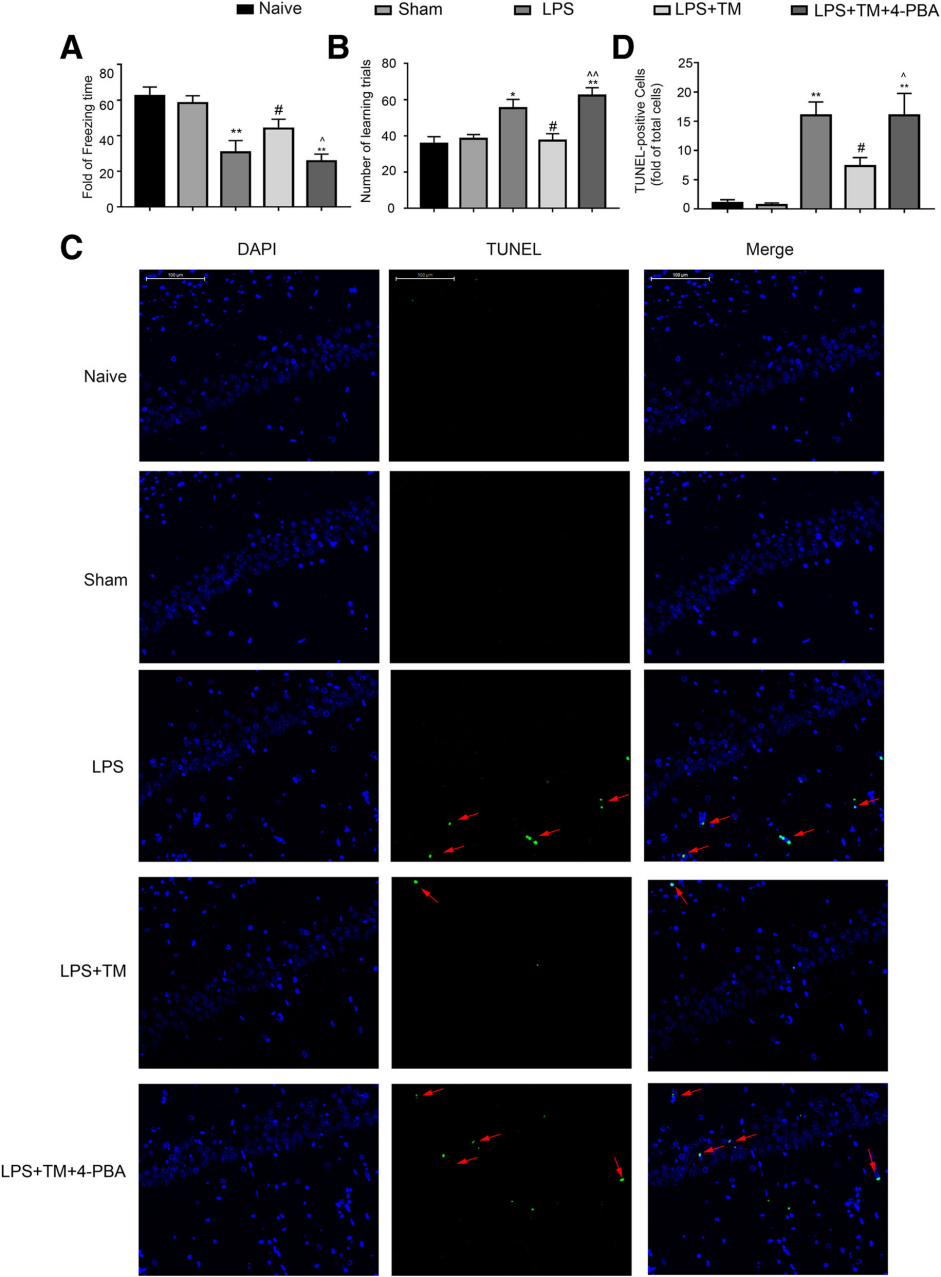
Mild ER stress inhibited LPS-induced apoptosis in the hippocampus and ameliorated the cognitive decline. **a** Contextual fear response, as measured by freezing time, was determined in the rats (*n* = 12). **b** The number of learning trials was recorded to analyze the Y-maze test (*n* = 12). Each value was expressed relative to that of the naïve group, which was set to 100 (*n* = 6). **c** The TUNEL assay was performed to determine the extent of apoptosis in the CA1 area of the hippocampus. The arrows indicate cells showing an overlay of TUNEL and DAPI signals. Scale bar, 100 μm. **d** Quantitative analysis of TUNEL-positive cell content in different groups. The data are representative of three independent experiments. *P < 0.05, **P < 0.01 vs. naïve group. #*P* < 0.05, ##*P* < 0.01 vs. LPS treatment group. ^*P* < 0.05, ^^*P* < 0.01 vs. TM treatment group. The data are presented as the mean ± SEM

One day after LPS systemic injection, we performed a TFC assessment and the Y-maze test to observe the cognitive function of the rats. As shown in Fig. 4a, b, compared to the naïve group, rats exposed to LPS exhibited a significant reduction in cognitive function. Notably, treatment with TM (3 μg) significantly improved the freezing behavior and the number of learning trials, indicating TM may help protect against the memory dysfunction caused by LPS.

#### TM alleviated LPS-induced neuronal apoptosis

To determine whether TM affected neuronal apoptosis, we performed TUNEL assay in the hippocampal CA1 region (Fig. 4c).

There was no TUNEL staining observed in the hippocampus of the naïve group, while TUNEL-positive cells were abundant in the hippocampus of LPS group.TM administration induced a dramatic decrease in TUNEL-positive cells in the hippocampal CA1 region (Fig. 4c, d). These data suggested a protective role of TM in LPS-induced neuroinflammation.

#### 4-PBA reversed the neuroprotective effects of TM

To clarify whether mild ER stress activation is responsible for the neuroprotection of TM, rats were administered 4-PBA (a chemical chaperone known to reduce ER stress) at a dosage of 100 mg/kg. Additionally, it has been reported that this dose does not affect the normal function of the nervous system [35, 36].

**Fig. 5:**
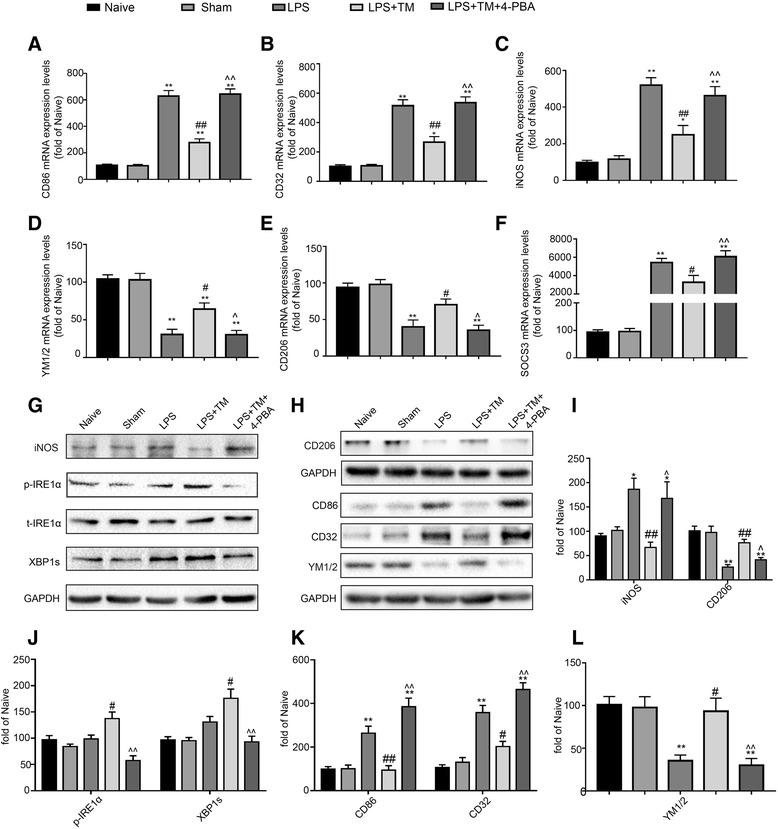
Mild ER stress attenuated LPS-induced neuroinflammation and shifted microglia polarization from M1/2b to M2a. **a**–**c** Expression levels of M1 phenotype markers (CD86, CD32, and iNOS). **d**, **e** Expression levels of markers for the M2a phenotype (YM1/2 and CD206). **f** Expression levels of SOCS3, a marker for the M2b phenotype. The expression levels of iNOS, CD206, p-IRE1α, and XBP1s were detected in the hippocampus of rats by Western blotting using specific antibodies. Each blot is representative of three experiments. **g**–**l** Expression of p-IRE1α was quantified and normalized to t-IRE1α levels, and the expression levels of iNOS, CD206, XBP1s, CD86, CD32, and YM1/2 were quantified and normalized to GAPDH levels. Each value was expressed relative to the values of the naïve group, which was set to 100 (*n* = 6). The data are representative of three independent experiments. *P < 0.05, **P < 0.01 vs. naïve group. #*P* < 0.05, ##*P* < 0.01 vs. LPS treatment group. ^*P* < 0.05, ^^*P* < 0.01 vs. TM treatment group. The data are presented as the mean ± SEM

Treatment with 4-PBA decreased ER stress, as demonstrated by reduced p-IRE1α and XBP1s protein levels compared with levels in the non-PBA treated group (Fig. 5g). Importantly, concomitant administration of 4-PBA and TM partially blocked the neuroprotection conferred by TM as reflected by the increased expression of TUNEL-positive cells and a significant reduction in cognitive function, while 4-PBA alone was without effects in healthy rats (Additional file 2: Figure S1). Therefore, these results further confirmed that low doses of TM may help protect against LPS-induced cognitive dysfunction and inhibit caspase-3 activation by (at least partially) inducing mild ER stress.

### Mild ER stress attenuated LPS-induced neuroinflammation and shifted the microglia population from M1/2b to M2a microglia in the hippocampus

#### TM reversed LPS-induced M1/2b microglia activation genes in the hippocampus

Because microglia activation is an early sign that triggers neuronal death in neurodegenerative disorders, we explored the effect of mild ER stress on microglia in vitro [41]. We first measured the expression of microglia genes associated with a classic (M1), alternative repair and regeneration (M2a), and immunomodulatory (M2b) phenotypes based on identifying antigenic markers for each microglial state.

Figure 5a–c shows the relative mRNA expression of the typical M1 genes CD86, CD32, and inducible nitric oxide synthase (iNOS). The M2a genes YM1/2 and CD206 and the M2b gene suppressor of cytokine signaling3 (SOCS3) were the other markers selected in the present study (Fig. 5d–f).

LPS significantly increased the mRNA levels of M1 and M2a markers in the hippocampus compared with the levels observed in the naïve group. In contrast, the M2a-repair/regeneration marker genes were significantly reduced compared with those in the naïve group. TM pretreatment markedly altered the balance of M1 and M2 microglia expression patterns in the hippocampus with significantly increased expression of M2a genes and decreased expression of several key M1 and M2b genes.

Western blotting also showed that the expression of iNOS, CD86, and CD32 in the hippocampi of LPS-injected rats was significantly higher than that in the naïve rats, but these elevations were remarkably inhibited by TM pretreatment. The expression of CD206 and YM1/2 was decreased in LPS-treated rats, while TM reversed the LPS-induced reduction of CD206 and YM1/2 levels (Fig. 5g–l).

#### TM inhibited iNOS expression and increased the expression of CD206 in LPS-injected rats

**Fig. 6:**
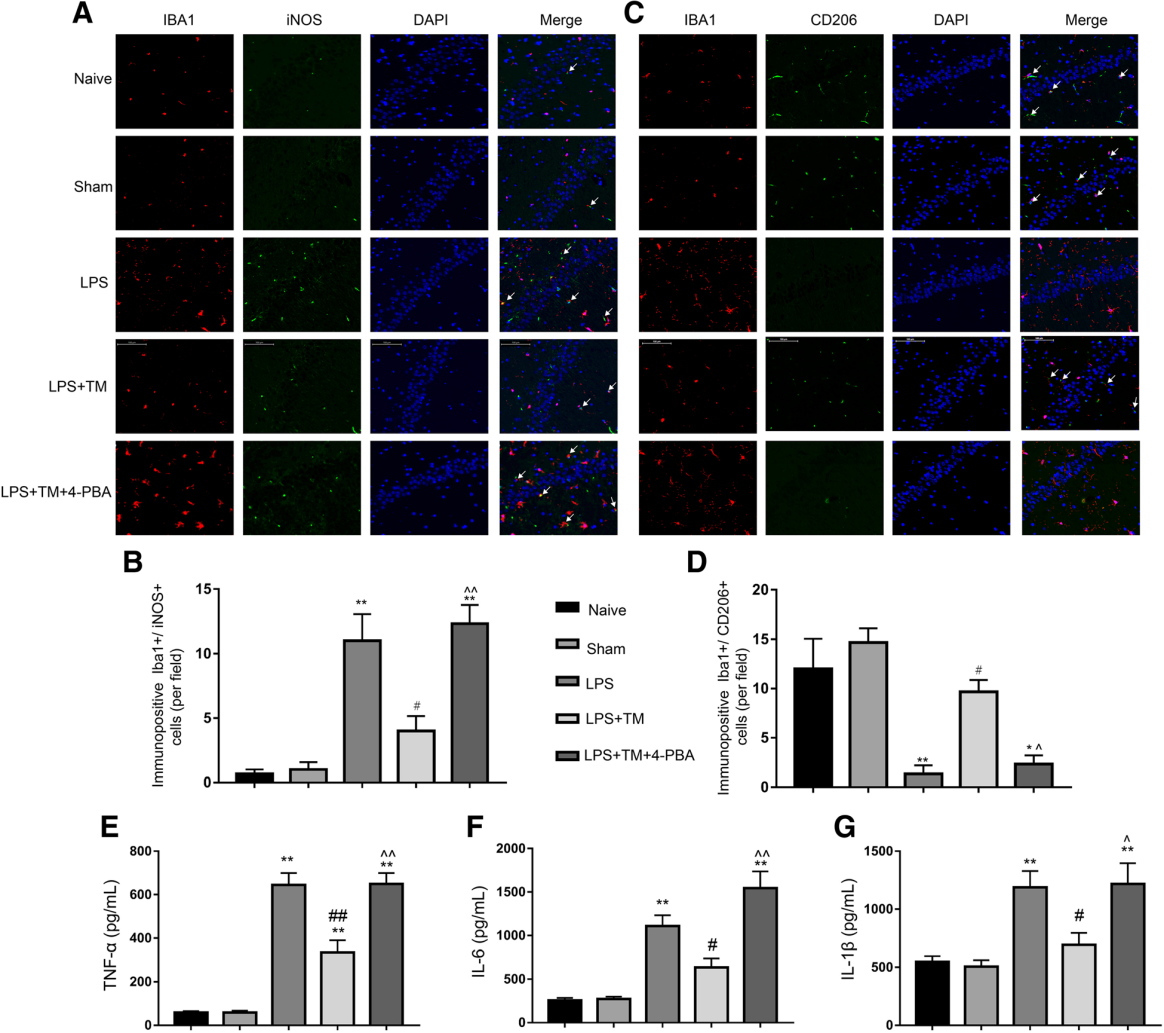
Mild ER stress contributed to M2a polarization in hippocampal CA1. **a**, **b** Double-staining immunofluorescence of IBA1 with either iNOS or CD206 in the CA1 area of the hippocampus (indicated by arrows). Scale bars, 100 μm. **c** Quantification of IBA1^+^/ iNOS^+^ double-stained cells in the hippocampal CA1. **d** Quantification of IBA1^+^/CD206^+^ double-stained cells in the hippocampal CA1. **e**, **f**, **g**. The levels of the proinflammatory factors TNF-α, IL-6, and IL-1β were detected by ELISA (n=5). The data are representative of three independent experiments. *n* = 6. *P < 0.05, **P < 0.01 vs. naïve group. #*P* < 0.05, ##*P* < 0.01 vs. LPS treatment group. ^*P* < 0.05, ^^*P* < 0.01 vs. TM treatment group. The data are presented as the mean ± SEM

To further evaluate whether microglia polarized in a particular manner, representative M1-associated (iNOS) or M2-associated (CD206) marker proteins were analyzed by double immunofluorescent staining with the microglia marker Iba1 in hippocampal CA1 region (Fig. 6a, b). Consistent with Western blotting results in Fig. 5g, a significant decrease of the M1 marker iNOS expression was observed after TM administration in Iba1^+^ microglia compared with microglia in the LPS group (Fig. 6a, b). In contrast, immunofluorescence for the M2 marker CD206 was significantly decreased in Iba1^+^ cells treated with LPS compared to the levels in the control group; however, this decrease was abolished after TM treatment (Fig. 6c, d). These results indicated that TM could inhibit LPS-induced microglia activation and shift the phenotype of microglia towards M2a.

#### TM inhibited the hippocampal proinflammatory cytokines secretion induced by LPS

Because neuroinflammation is mainly due to the excessive secretion of proinflammatory factors, the levels of TNF-α, IL-1β, and IL-6 were detected by ELISA. LPS induced significant increases in the production of proinflammatory factors, and TM (3 μg) could inhibit this inflammatory response (Fig. 6e–g).

#### 4-PBA attenuated the anti-neuroinflammatory effects of TM in LPS-treated rats

To further clarify whether mild ER stress is involved in the anti-neuroinflammatory properties of TM, ER stress was blocked using 4-PBA. When 4-PBA was given to healthy rats alone, there was no obvious proinflammatory effect (Additional file 2). However, the expression levels of TNF-α, IL-1β, and IL-6 were markedly enhanced after 4-PBA co-treatment, leading to the significant reversal of the anti-neuroinflammatory effects of TM (Fig. 6e–g). Moreover, although TM reversed the LPS-induced M1/M2 imbalance, 4-PBA greatly diminished these effects. Therefore, these results indicated that mild ER stress may be required for TM-induced neuroprotection.

### Low doses of TM caused a nontoxic, mild UPR in primary cultured microglia

**Fig. 7:**
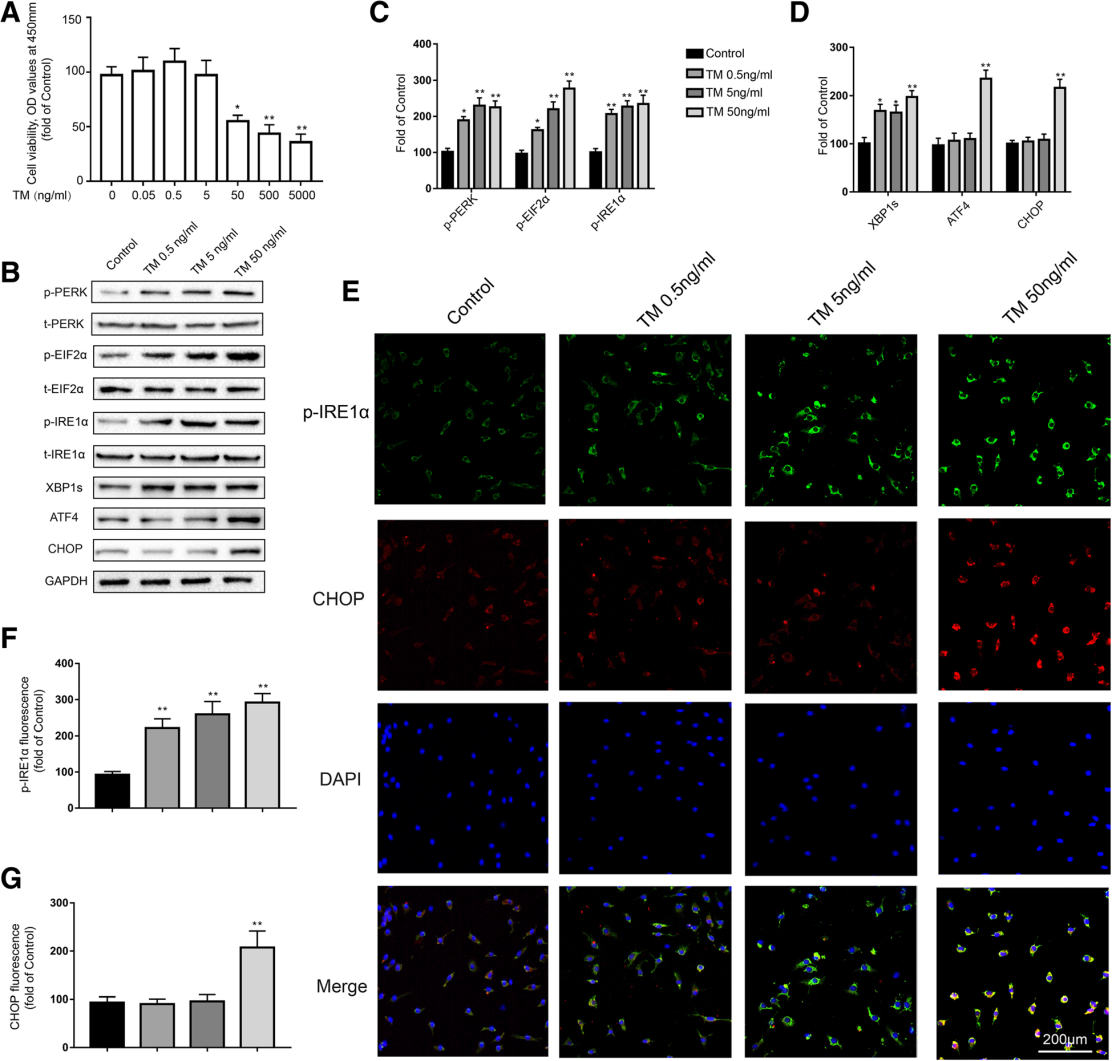
Low doses of TM activate a nontoxic, mild UPR in primary cultured microglia. **a** Primary microglia were treated with TM (0.02 to 2000 ng/ml) for 24 h followed by assessment of cell viability using the CCK-8 assay. **b** The expression levels of p-PERK, p-EIF2α, p-IRE1α, XBP1s, ATF4, and CHOP in primary microglia were detected by Western blotting using specific antibodies. Each blot is representative of three experiments. **c** Phosphorylated levels of PERK, EIF2α, and IRE1α were quantified and normalized to corresponding total levels. **d** The expression levels of XBP1s, ATF4, and CHOP were quantified and normalized to GAPDH levels. **e** Cells were stained with p-IRE1α and CHOP antibodies. p-IRE1α-immunopositive (green) and CHOP-immunopositive (red) expression in primary microglia was observed using confocal scanning. Blue staining represents DAPI. Scale bar, 200 μm. **f**, **g** Quantitative data of the mean intensity of p-IRE1α and CHOP fluorescence in primary microglia. Each value was then expressed relative to that of the naïve group, which was set to 100. All experiments were repeated three times. *P < 0.05, **P < 0.01 vs. naïve group. The data are presented as the mean ± SEM

Multiple investigations have shown that multiple cell types are vulnerable to ER stress-induced cell death. Therefore, primary rat microglia were first subjected to several concentrations of TM for 24 h and assayed for cell viability using the CCK-8 reagent. Our results indicated that compared with the control treatment, TM (< 50 ng/ml) did not affect the viability of primary rat microglia (Fig. 7a). Therefore, 0.5, 5, and 50 ng/ml were selected for subsequent experiments.

In light of our findings in vivo, we first investigated whether low doses of TM could also activate a mild UPR in primary cultured microglia. Compared with the control group, doses of 0.5, 5, and 50 ng/ml TM increased the protein levels of p-IRE1α, XBP1s, p-PERK, and p-EIF2α (Fig. 7b, c). It is worth noting that TM at 50 ng/ml triggered a robust UPR as evident by the significant increase in the protein levels of ATF4 and CHOP, while TM at 0.5 and 5 ng/ml had no significant influence on the expression levels of ATF4 and CHOP (Fig. 7d). Similar results were also observed in the immunofluorescence assay (Fig. 7e). Immunofluorescent analysis showed that TM (0.5, 5, and 50 ng/ml) induced an increase in p-IRE1α expression that was significantly higher than that in the control group (Fig. 7f). CHOP expression was not altered in primary cultured microglia treated with TM at 0.5 or 5 ng/ml. Interestingly, 50 ng/ml TM promoted the expression of CHOP (Fig. 7g). These results demonstrated that low doses of TM (0.5 and 5 ng/ml) could also cause nontoxic, mild ER stress in primary cultured microglia.

### Mild ER stress inhibited cytokine production and promoted M2a polarization in primary cultured microglia

To validate the in vivo findings, we tested whether mild ER stress-mediated neuroprotection is effective in vitro. According to the results above, we chose a dose of TM (5 ng/ml) to induce mild ER stress in microglia.

#### TM inhibited proinflammatory cytokine production in primary cultured microglia

**Fig. 8:**
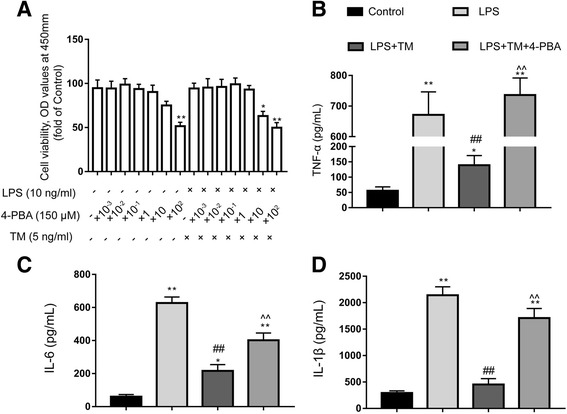
Mild ER stress inhibits proinflammatory cytokine production in primary cultured microglia. **a** Primary cultured microglia were subjected to LPS and TM and treated with the indicated dosage of 4-PBA. After 24 h of treatment, cell viability was determined using CCK-8. **b**–**d** The levels of the proinflammatory factors TNF-α, IL-6, and IL-1β were detected by ELISA. The data are representative of three independent experiments. *P < 0.05, **P < 0.01 vs. naïve group. #*P* < 0.05, ##*P* < 0.01 vs. LPS treatment group. ^*P* < 0.05, ^^*P* < 0.01 vs. TM treatment group. The data are presented as the mean ± SEM

Microglia were treated with TM for 1 h followed by LPS (10 ng/ml) stimulation for 24 h, and the proinflammatory cytokine levels were analyzed by ELISA. As the results show in Fig. 8a, at 24 h after LPS administration, no visible alterations in cell viability were detected by CCK-8. However, LPS triggered increases in the IL-1β, IL-6, and TNF-α levels, which could be partially reversed by 5 ng/ml TM (Fig. 8b–d). These results suggested that TM protected microglia from LPS stimulation.

#### TM induced microglial changes in polarization from M1/2b to M2a

**Fig. 9:**
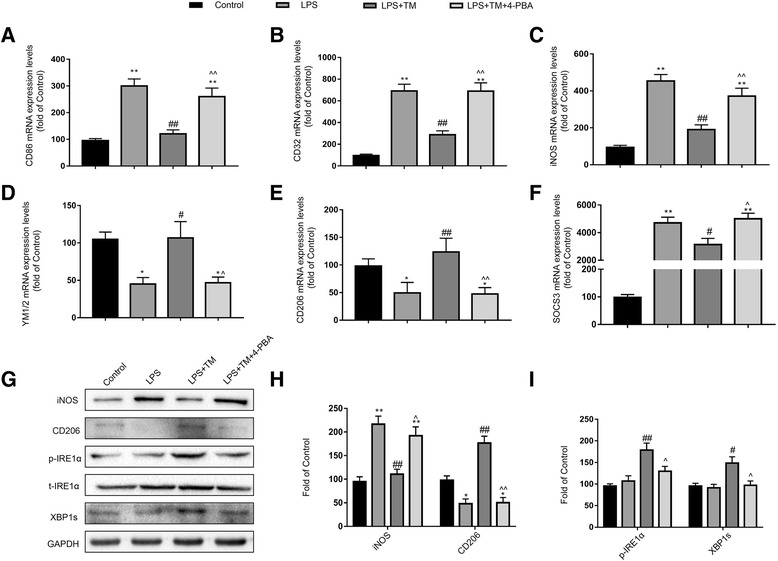
Mild ER stress induces microglial changes in polarization from M1/2b to M2a. **a**–**c** Expression levels of M1 phenotype markers (CD86, CD32, and iNOS). **d**, **e** Expression levels of M2a phenotype markers (YM1/2 and CD206). **f** Expression levels of SOCS3, a marker for the M2b phenotype. The expression levels of iNOS, CD206, p-IRE1α, and XBP1s were detected by Western blotting using specific antibodies in the primary microglia. Each blot is representative of three experiments. **g**, **h**, **i** Expression of p-IRE1α was quantified and normalized to t-IRE1α levels, and the expression levels of iNOS, CD206, and XBP1s were quantified and normalized to GAPDH levels. Each value is expressed relative to that in the control group, which was set to 100. All experiments were repeated three times. *P < 0.05, **P < 0.01 vs. naïve group. #*P* < 0.05, ##*P* < 0.01 vs. LPS treatment group. ^*P* < 0.05, ^^*P* < 0.01 vs. TM treatment group. The data are presented as the mean ± SEM

**Fig. 10:**
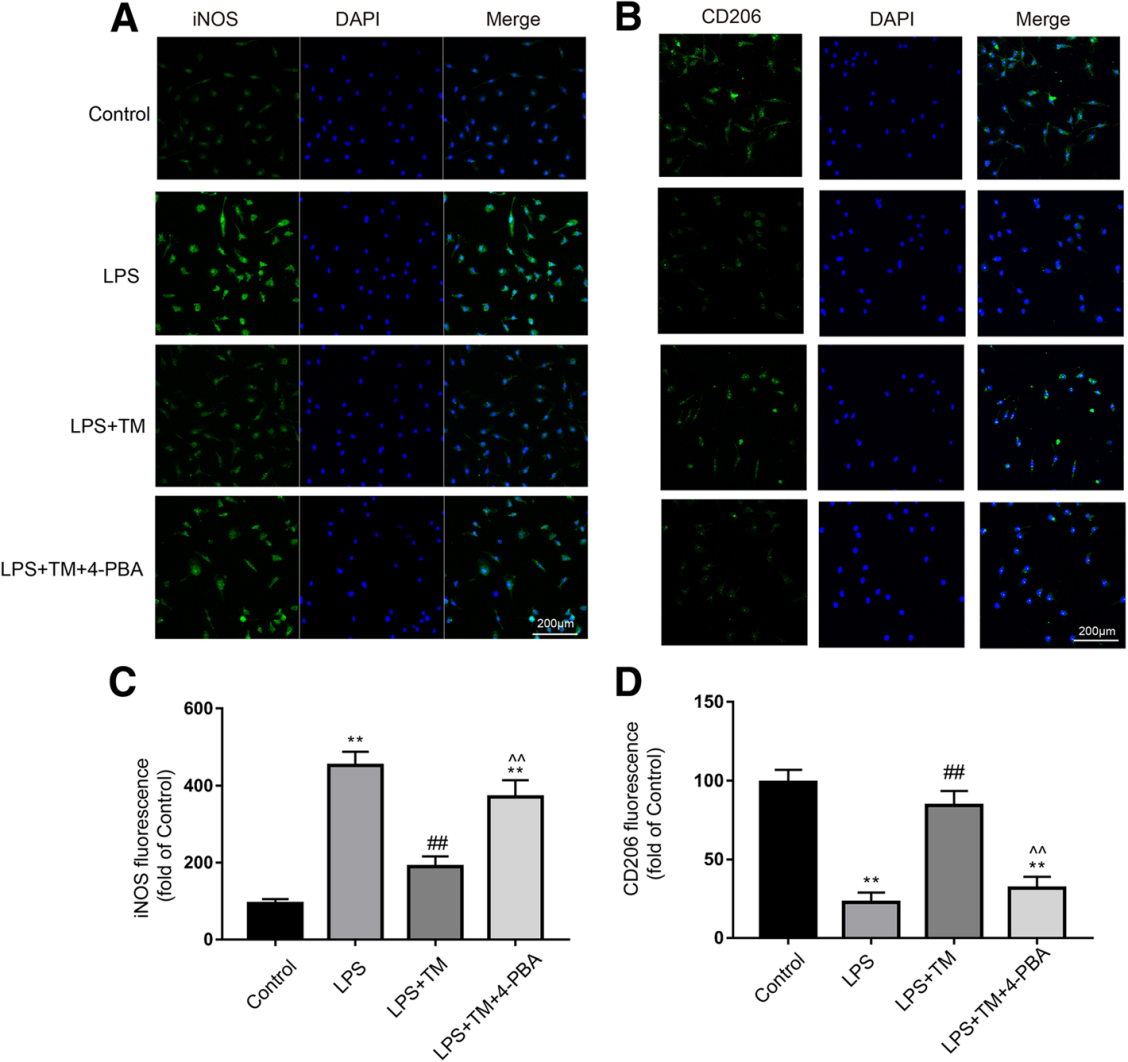
Mild ER stress contributed to M2a polarization of microglia. **a**, **b** Cells were stained with an iNOS or CD206 antibody as indicated. Blue staining represents DAPI. Scale bar, 200 μm. **c**, **d** Quantitative data of the mean intensity of iNOS and CD206 fluorescence in primary microglia. Each value is expressed relative to that in the control group, which was set to 100. All experiments were repeated three times. *P < 0.05, **P < 0.01 vs. naïve group. #*P* < 0.05, ##*P* < 0.01 vs. LPS treatment group. ^*P* < 0.05, ^^*P* < 0.01 vs. TM treatment group. The data are presented as the mean ± SEM

To further confirm the effects of TM on the phenotype changes of primary microglia, markers corresponding to the different microglia phenotypes were tested. LPS increased the expression of M1 markers (CD86, CD32, iNOS) and the immunomodulatory M2b marker SOCS3 and caused a decrease in the expression of M2a-repair/regeneration markers (YM1/2 and CD206). Pretreatment with TM (5 ng/ml) for 1 h can effectively shift the phenotypes of microglia towards M2a (Fig. 9a–f). In addition, microglia were labeled with iNOS (a typical M1 marker) and CD206 (a typical M2a marker) by immunofluorescence and Western blot analysis, which also confirmed the phenotype changes in the primary microglia in response to TM treatment. As the results in Fig. 10a–d, TM preconditioning decreased iNOS expression and increased of CD206 expression compared to the LPS group. These data indicated that TM reversed the LPS-induced imbalance of microglial M1/M2 polarization.

#### 4-PBA impaired the anti-inflammatory effects and M2a differentiation conferred by TM

Next, we examined whether TM-mediated M2a differentiation and anti-inflammatory response in LPS-stimulated microglia required mild ER stress. To achieve this goal, we blocked ER stress using 4-PBA. Microglia were first subjected to TM (5 ng/ml) with or without 4-PBA at the indicated concentrations for 1 h followed by stimulation with LPS (10 ng/ml) for 24 h. As shown in Fig. 8a, our results indicated that 4-PBA (< 1500 μM) in the presence of TM (5 ng/ml) and 10 ng/ml LPS exerted no obvious toxic effects on microglia. Therefore, we chose the dose of 150 μM 4-PBA to manipulate the UPR in microglia.

Microglia were pre-treated with 4-PBA (150 μM) and TM (5 ng/ml) for 1 h. Cells were then treated with LPS for 24 h. As shown in Fig. 9g, the effects of 4-PBA against mild ER stress were efficient as it strongly reduced p-IRE1α and XBP1s protein expression compared with that in non-PBA treated cells (Fig. 9g–i). Similar to the results in vivo, 4-PBA almost completely blocked the TM-mediated anti-inflammatory effects and M2a differentiation (Figs. 8b–d and 9a–f). We wondered whether this effect of 4-PBA resulted from the induction of cell toxicity. Using CCK-8 assays, we ruled out this possibility because co-incubation with 4-PBA (150 μM), TM (5 ng/ml), and LPS (10 ng/ml) for 24 h did not significantly affect cell viability (Fig. 8a). Altogether, these results demonstrate that mild ER stress is required for TM-mediated protection in primary cultured microglia.

## Discussion

With the increased life expectancy in recent decades, neurodegenerative diseases have become a large health and economic burden. Neuroinflammation has emerged as a prominent component in the pathology of many neurodegenerative diseases, including AD and PD [1, 2]. Although there are many active efforts to understand the mechanisms responsible for neuroinflammatory regulation in neurodegenerative diseases, the mechanisms underlying how neuroinflammation in the central nervous system (CNS) is regulated have not been identified. In the present study, we showed that mild ER stress played a key role in regulating neuroinflammation in the CNS.

The occurrence of ER stress in CNS may represent (1) a mild and pro-survival response which can engage adaptive stress signaling events and re-establish protein homeostasis, (2) a severe and irreversible damaging process triggering neuronal damage, or (3) a late epiphenomenon due to irreversible and untreatable alterations in homeostasis [42]. ER stress engages a signal transduction pathway called the unfolded protein responses (UPR). The mammalian cell senses the accumulation of unfolded proteins by three key ER resident proximal sensors including PERK, IRE1α, and ATF6 [10, 40].

Much of our understanding of the UPR, including its role and regulation in neuroinflammation, comes from studies that utilize severe pharmacological perturbation of experimental animals or cultured cells. However, these studies almost certainly fail to recapitulate the UPR as it is elicited under the conditions of milder ER stress that could be encountered physiologically. It is becoming clear that the UPR activation can either enhance or reduce neuroinflammation and sometimes may even have opposing effects on pathological conditions depending upon the extent of ER stress [7, 23, 24]. Thus, one of the fundamental questions of the UPR is whether the response can allow experimental animals to adapt to neuroinflammation and escape neuroinflammation-induced memory deficiency.

In the present investigation, we found that ER stress inducer TM at a low dosage produced mild perturbations of ER function and led to a modest UPR. Despite this modest effect, a high concentration of TM caused serious ER perturbation and a robust UPR. Our data demonstrated that activation of a moderate UPR did not induce microglia or hippocampal lethality but rather promoted an adaptive response that protected against LPS-stimulated neuroinflammation and memory deficiency. 4-PBA, a compound that alleviates ER stress, counteracted the neuroprotection of TM; this activity also supported a protective role of mild UPR against neuroinflammation.

One possible mechanism to explain neuroprotection during mild ER stress would be selective activation of one or more of the proximal sensors of ER stress. The ER sensor proteins including PERK and IRE1α are responsible for both the adaptive and the proapoptotic pathways of UPR. In a recent study, it was shown that moderate activation of the PERK–EIF2α pathway provides cell survival signals, possibly via a reduction in the load of unfolded proteins in the ER [19]. Furthermore, the spliced form of XBP1 was previously shown to prevent β-amyloid neurotoxicity [17, 18], suggesting that selective activation of the IRE1α-XBP1s arm of the UPR may decrease the progression of AD. To pursue this idea, we monitored the activation of IRE1α-XBP1s, and PERK-eIF2α signaling in response to varying concentrations of TM. However, our data supported the notion that both high and low concentrations of TM activated PERK and IRE1α pathways and that selective activation of proximal stress signaling molecules was not required for neuroprotection.

Another way in which neuroprotection could be favored as an outcome is if a mild UPR diverges from a severe UPR in the expression of downstream genes. A paradox of the UPR is that the response leads to the simultaneous activation of both protective and pro-apoptotic pathways. The best characterized of these pro-apoptotic pathways is production of the CHOP, which is regulated by ATF4 [43]. To test this hypothesis, we performed TUNEL assay to determine the extent of apoptosis, and monitored the expression of ATF4 and CHOP protein, in response to varying concentrations of TM.

For TM treatment, we found that, although low concentrations of TM resulted in the induction of the PERK–EIF2α/IRE1-XBP1pathway, ATF4/CHOP upregulation was lost by 24 h of treatment under conditions that the UPR allowed for neuroprotection, but not at higher concentrations that promoted neuroinflammation and apoptosis. We demonstrated that protection to neuroinflammation was an intrinsic consequence of nontoxic, mild activation of the UPR in rat hippocampus and primary microglia, and was accompanied by changes in the expression of downstream proteins that were qualitatively distinct from the UPR as induced by severe ER stress. These data are consistent with the notion that the proximal sensors of ER stress are activated by mild ER stress, but that there is a divergence in the expression of downstream proteins depending upon whether the outcome is protective or apoptosis.

Mild ER stress-mediated protective effects can be assimilated as pre-conditioning or an adaptive stress response, also termed hormesis. Hormesis refers to a biological protective response induced by a low (or mild) exposure of toxins and other stressors [25, 27]. Conditions that stimulate hormesis can allow an organism or cell to better respond to a high dose of a second stimulus [23]. In medicine, quick cycles of myocardial ischemic preconditioning prepare the heart before surgery. Several studies have proposed that mild ER stress also induces a hormetic response called ER hormesis [12, 22]. This concept has also been applied to the medical field. Previous studies found that pharmacological pretreatment with a nonlethal dose of ER inducers promoted neuroprotection in Drosophila and mouse models of Parkinson’s disease [15]. Preconditioning with low levels of ER stress alleviated spinal cord injury [44, 45], brain inflammation [46, 47], and brain ischemia/reperfusion [46, 47].

Our work is an example of ER-mediated hormesis (or ER hormesis) too. When the ER stress sensed by the cell in the CNS is milder, the overall level of activation of the UPR is considerably less. Because ATF4 and CHOP are quite unstable at both the mRNA and protein levels, changes in the expression of these proteins are necessarily short-lived in the absence of a positively robust ER stress signal. Therefore, mild perturbations of ER function may actually promote a hormetic mechanism of protection against subsequent and stronger pathological stimuli in the long term. Nevertheless, when the ER stress is robust and persistent, ER hormetic adaptive mechanisms is insufficient to thwart the pro-apoptotic program. CHOP, an important proapoptotic protein in its own right, and also presumably a sentinel for the activation of pro-apoptotic cascades in general, is expressed at levels too high to be downregulated, and ER-related neurotoxicity occurs as a consequence. However, the molecular mechanisms that facilitate the switch from protection to impairment are still poorly understood.

As cells that populate the parenchyma of the CNS, microglia can constantly sense the extracellular environment. Activation of microglia has been demonstrated to be an early sign that often precedes and triggers further neuroinflammatory processes leading to the exacerbation of neurotoxicity in neurodegenerative diseases [3, 4]. Microglia can exert dual effects dependent on their intrinsic properties, interaction with environmental pathogenic factors, and composition of the cellular microenvironment. Microglia can be characterized into three main states: a classical “M1” reactive phenotype with neurotoxic properties, an alternative “M2a” phenotype with an alternate activation that is involved in repair and regeneration, and an M2b phenotype with immunoregulatory activity [41]. Cell differentiation entails an increase in protein synthesis that primes cells for ER stress and UPR activation. Thus, ER stress contributes to cell differentiation, such as CD8^+^ T cells, plasma cell, and B lymphocyte [7]. Although the underlying mechanisms still require systematic investigation, our initial results described here indicated that mild ER stress induced M2a microglial differentiation. Considering the contribution of the M2a phenotype to neuroprotection, shifting differentiated microglia from the M1/2b towards M2a phenotype via mild ER stress represents a novel therapeutic approach to treating neurodegenerative diseases and cognitive impairment.

Perhaps the most illuminating result of our work is that it suggests a rationale to explain how the ER stress can be a predominantly protective pathway under certain circumstances. The central feature of a protective response to ER stress appears to be maintenance of expression of proteins that facilitate survival, without persistence of pro-apoptotic proteins such as CHOP and ATF4 [14].

## Conclusions

In summary, our results suggested that preconditioning with low levels of ER stress (i.e., exposure to nonlethal doses of pharmacological ER stressors such as TM) alleviated LPS-induced neuroinflammation and cognitive impairment. Our study proposed a new therapeutic possibility to trigger and maintain ER stress at a moderate level such that the stress response protects against or suspends the onset of neurodegenerative diseases or delays disease progression. Our study might provide not only advanced understanding of the actions of ER stress in neurodegeneration but also a rationale to define a novel target for neurodegeneration. Of course, there are many questions that need to be addressed in future studies.

## Additional files

Additional file 1: Supplemental materials and methods. (DOCX 24 kb)

Additional file 2: 4-PBA (100 mg/kg) had no neurotoxicity and proinflammatory effect in healthy rats. (DOC 621 kb)

## Abbreviations

4-PBA: 4-Phenylbutyric acid
AD: Alzheimer’s disease
ALS: Amyotrophic lateral sclerosis
ATF: Activating transcription factor
cck8: The Cell Counting Kit-8
CHOP: CCAAT/enhancer-binding protein-homologous protein
CNS: Central nervous system
DAPI: Fluoroshield mounting medium with 4,6-diamidino-2-phenylindole
DMEM: Dulbecco’s modified Eagle’s medium
DMSO: Dimethyl sulfoxide
EIF2α: Eukaryotic translation initiation factor 2α
ELISA: Enzyme-linked immunosorbent assay
ER stress: Endoplasmic reticulum stress
ip: Intraperitoneally
FCS: Fetal calf serum
HD: Huntington’s disease
icv: Intracerebroventricular injection
IL-1β: Interleukin-1β
IL-6: Interleukin-6
iNOS: Inducible nitric oxide synthase
IRE1α: Inositol-requiring protein 1α
LPS: Lipopolysaccharide
MS: Multiple sclerosis
PD: Parkinson’s disease
PERK: Total protein kinase RNA-like ER kinase
PMSF: Phenylmethylsulfonylfluoride
PrDs: Prion-related diseases
TBST: Tris-buffered saline with Tween 20
TFC: Trace fear conditioning
TM: Tunicamycin
TNF-α: Tumor necrosis factor-α
UPR: The unfolded protein response
XBP1s: Spliced X-box-binding protein-1
