# Correction to: Mild endoplasmic reticulum stress ameliorates lipopolysaccharide-induced neuroinflammation and cognitive impairment via regulation of microglial polarization

**DOI:** 10.1186/s12974-020-01990-3

**Published:** 2020-11-24

**Authors:** Yi-wei Wang, Qin Zhou, Xiang Zhang, Qing-qing Qian, Jia-wen Xu, Peng-fei Ni, Yan-ning Qian

**Affiliations:** 1 Department of Anesthesiology, The First Affiliated Hospital of NanjingMedical University, Nanjing, Jiangsu 210029 People’s Republic of China; 2 Department of Anesthesiology, Shanghai General Hospital, Shanghai JiaoTong University School of Medicine, Shanghai, 200080 People’s Republic of China

**Correction to: J Neuroinflammation (2017) 14:233**

**https://doi.org/10.1186/s12974-017-1002-7**

Following publication of the original article [1], the authors noticed a mistake on Fig. 3e and would like to correct it. Presented here is the corrected version of Fig. 3.

**Fig. 3:**
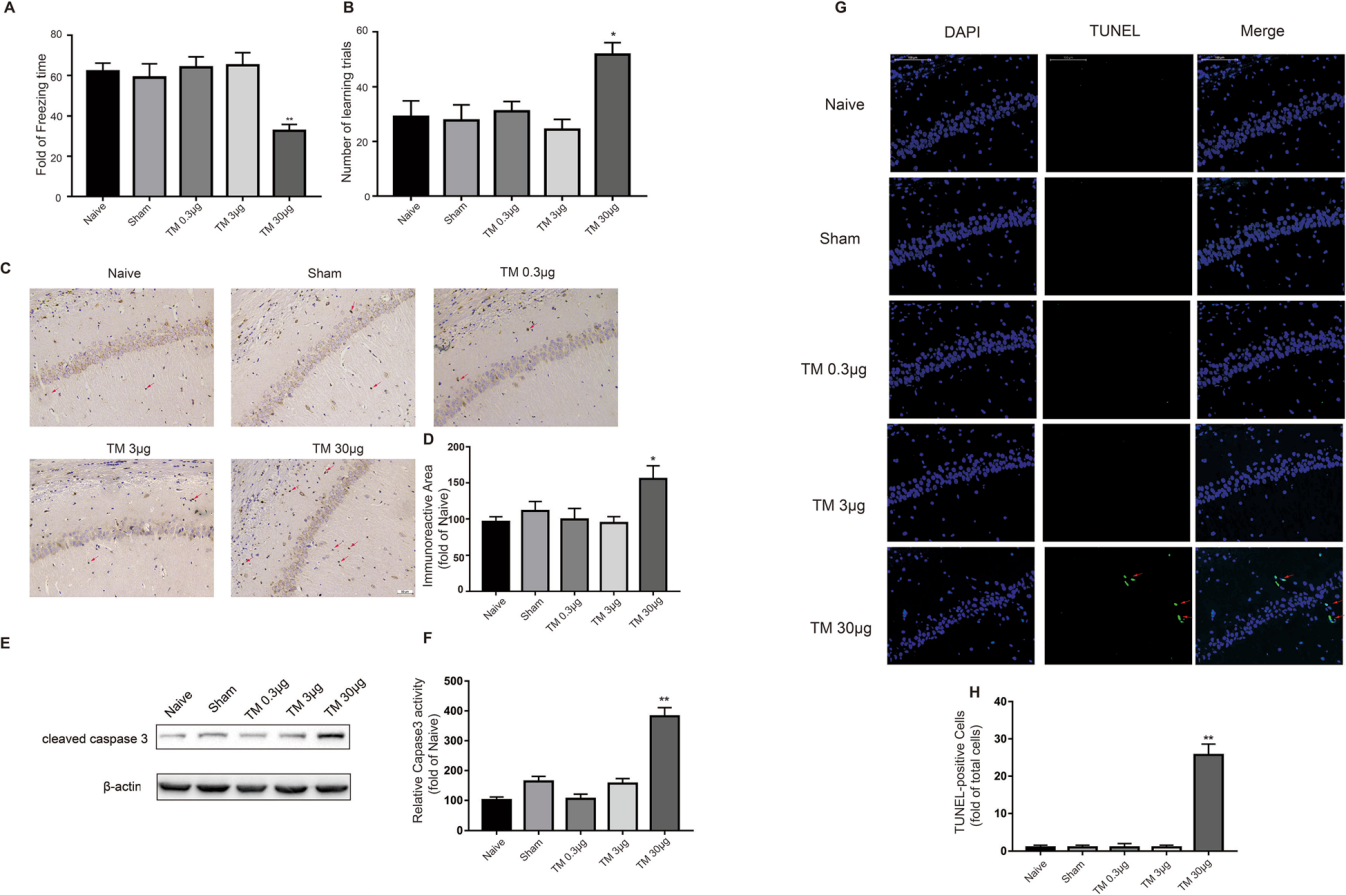
Low doses of TM activated a nonharmful, moderate UPR in the hippocampus. The freezing time in the trace fear conditioning test (**a**) and the number of learning trials in the Y-maze test (**b**) were recorded to analyze cognitive changes (*n* = 12). **c** Immunostaining was used to detect cleaved caspase-3 in the CA1 area of the hippocampus. Scale bar, 50 μm. **d** Quantification of cleaved caspase-3-positive cells in the CA1 area of the hippocampus. **e** The expression levels of cleaved caspase 3 in the hippocampus of rats were detected by Western blotting using specific antibodies. **f** Quantification of cleaved caspase-3-positive cells in the CA1 area of the hippocampus. Each value was expressed relative to that in the naïve group, which was set to 100 (*n* = 6). **g** The TUNEL assay was performed to determine the extent of apoptosis in the CA1 area of the hippocampus. The arrows indicate cells showing an overlay of TUNEL and DAPI signals. Scale bar, 100 μm. **h** Quantitative analysis of TUNEL-positive cell content in different groups. ^*^P < 0.05, ^**^P < 0.01 vs. naïve group. The data are presented as the mean ± SEM
