# Supplementary material for: Mild endoplasmic reticulum stress ameliorates lipopolysaccharide-induced neuroinflammation and cognitive impairment via regulation of microglial polarization
Source: J Neuroinflammation. 2017 Nov 28;14:233. doi: 10.1186/s12974-017-1002-7 (PMC5704515; doi:10.1186/s12974-017-1002-7)
Supplement: Supplementary file 1 — Supplemental materials and methods. (DOCX 24 kb) [file 12974_2017_1002_MOESM1_ESM.docx]

**Supplemental Materials and Methods**

**Reagents and antibodies**

Dulbecco’s modified Eagle’s medium (DMEM), 0.25% trypsin-EDTA solution and fetal calf serum (FCS) were purchased from Gibco-BRL (Grand Island, NY, USA). Tunicamycin (TM), 4-phenylbutyric acid (4-PBA), and lipopolysaccharide (LPS, from *Escherichia coli*, 0111:B4) were purchased from Sigma-Aldrich (St. Louis, MO, USA). Antibodies targeting CD206, total protein kinase RNA-like ER kinase (t-PERK), and phospho(T981)-PERK (p-PERK) were purchased from Santa Cruz Biotechnology (Santa Cruz, CA, USA). Antibodies against phosphor(S724)- inositol-requiring enzyme 1α (IRE1α), total IRE1α, iNOS, activating transcription factor (ATF)-4, CD86, CD32, YM1/2 and Fluoroshield Mounting Medium with 4’,6-diamidino-2-phenylindole (DAPI) were purchased from Abcam (Hong Kong, China). Antibodies against Iba-1 were purchased from Wako (Osaka, Japan). Antibodies against CCAAT/enhancer-binding protein-homologous protein (CHOP), total EIF2α (t-EIF2α), cleaved caspase-3, caspase-3, phosphor (S51)-EIF2α (p-EIF2α), spliced X-box binding protein-1 (XBP1s), XBP1u and goat anti-mouse secondary antibodies and goat anti-rabbit secondary antibodies were obtained from Cell Signaling Technology (Beverly, MA, USA). A rat interleukin-6 (IL-6) enzyme-linked immunosorbent assay (ELISA) kit, rat IL-1β ELISA kit, and rat tumor necrosis factor (TNF)-α ELISA kit were obtained from eBioscience (San Diego, USA). RIPA buffer, Cell Counting Kit-8 (CCK-8) kit and the BCA kit were obtained from Beyotime (Shanghai, China). The one-step TUNEL apoptosis assay kit is obtained from KeyGEN BioTECH (KGA7074).

**Behavioral tests**

**Trace fear conditioning (TFC)**

Hippocampal-dependent memory in rodents was evaluated by trace fear conditioning (TFC) as previously described ^1-3^. Rats were trained to associate an environment (context) with a conditional stimulus (tone) and an unconditional stimulus (foot shock). The training consisted of placing the rat in the conditioning chamber and allowing exploration of the surroundings for 100 s. Next, the conditional stimulus—an auditory cue (80 dB, 5 kHz)—was presented for 20 s. The unconditional stimulus, a 2-s foot shock (0.8 mA), was administered after termination of the tone. This procedure was repeated with an interval of 100 s, and the rats were removed from the chamber 30 s later. Contextual assessment was performed 24 h after surgery in the same chamber but with no cues (tone or shock). Freezing behavior, recognized as lack of movement, was recorded for 300 s by video and analyzed using software (Xeye Fcs, Beijing MacroAmbition S&T Development Co., Ltd., Beijing, China). A decrease in the percentage of time spent frozen indicated impairment of memory.

**Y-maze test**

The Y-maze test was used to assess spatial working memory in rodents as previously described ^4^. The Y-maze consisted of three identical arms (30 ×5 × 20 cm). Each arm had a lamp at the distal end. A safe region was associated with the illumination, whereas the other regions featured electrical foot stimulation (40 ± 5 V). One arm was randomly selected as the “start” arm. The rat was put into the end of the “start” arm (starting area chosen randomly) and allowed to explore the maze freely for 3 min. The test was then started, and the illuminated arm (safe region) served as the new starting area. Furthermore, we randomly changed the orientation of the safe and stimulation regions. The test was considered successful when all four paws of the rats reached the safe region within 10 s. After each foot stimulation, we waited for the rat to reach the illuminated arm (the new starting area) before the next stimulation. If nine responses were correct in ten consecutive foot stimulations (9/10 standard), the rats were classified as having functional working memory. The total number of stimulations to reach the criterion during training was recorded as the learning ability. All rats satisfied the learning criterion in the present study.

**Enzyme-linked immunosorbent assay (ELISA)**

Cytokine quantification in the hippocampus and microglia was assessed by ELISA using commercial kits for IL-1β, IL-6, IL-10 and TNF-α according with the manufacturer’s instructions. Briefly, 96-well microplates were sensitized with the primary antibody at room temperature (RT) for 30 min, and then the samples were added and incubated at 37°C for 30 min. After the samples were washed, secondary antibody conjugated with peroxidase was added and incubated. The cytokine concentrations were spectrometrically determined using a micro ELISA reader.

**RT-PCR primers**

Primers are listed as the following: CD86-forward, GACACCCACGGGATCAATTA; CD86-reverse,AGGTTTCGGGTATCCTTGCT;CD32- forward, AGTTCGTTGCCGGTATTGAC; CD32-reverse, TTCCCTGTGATCAGGGTTTC; CD206-forward, TGTGAGCAACCACTGGGTTA; CD206-reverse, GTGCATGTTTGGTTTGCATC; SOCS3-forward, CCTCTGAGGTTCAGGAGCAG; SOCS3-reverse, CGTTGACAGTCTTCCGACAA; β-actin-forward, GGGTGTGAACCACGAGAAAT;β-actin-reverse, CCACAGTCTTCTGAGTGGCA; iNOS-forward, TCCTCAGGCTTGGGTCTTGT; iNOS-reverse, AGAAACTTCCAGGGGCAAGC; Ym1/2-forward, CAGGGTAATGAGTGGGTTGG;Ym1/2-reverse, CACGGCACCTCCTAAATTGT;TNFα-forward, AAVACACGAGACGCTGAAGT; TNFα-reverse, TCCAGTGAGTTCCGAAAGCC.

**References:**

1. Feng X, Degos V, Koch LG et al. Surgery results in exaggerated and persistent cognitive decline in a rat model of the Metabolic Syndrome. ANESTHESIOLOGY. 2013;118:1098-1105.

2. Terrando N, Yang T, Ryu JK et al. Stimulation of the alpha7 nicotinic acetylcholine receptor protects against neuroinflammation after tibia fracture and endotoxemia in mice. Molecular medicine (Cambridge, Mass.). 2015;20:667-675.

3. Almolda B, de Labra C, Barrera I et al. Alterations in microglial phenotype and hippocampal neuronal function in transgenic mice with astrocyte-targeted production of interleukin-10. Brain, behavior, and immunity. 2015;45:80-97.

4. Lu SM, Yu CJ, Liu YH et al. S100A8 contributes to postoperative cognitive dysfunction in mice undergoing tibial fracture surgery by activating the TLR4/MyD88 pathway. BRAIN BEHAV IMMUN. 2015;44:221-234.
