# Supplementary material for: Mild endoplasmic reticulum stress ameliorates lipopolysaccharide-induced neuroinflammation and cognitive impairment via regulation of microglial polarization
Source: J Neuroinflammation. 2017 Nov 28;14:233. doi: 10.1186/s12974-017-1002-7 (PMC5704515; doi:10.1186/s12974-017-1002-7)
Supplement: Supplementary file 2 — 4-PBA (100 mg/kg) had no neurotoxicity and proinflammatory effect in healthy rats. (DOC 621 kb) [file 12974_2017_1002_MOESM2_ESM.doc]

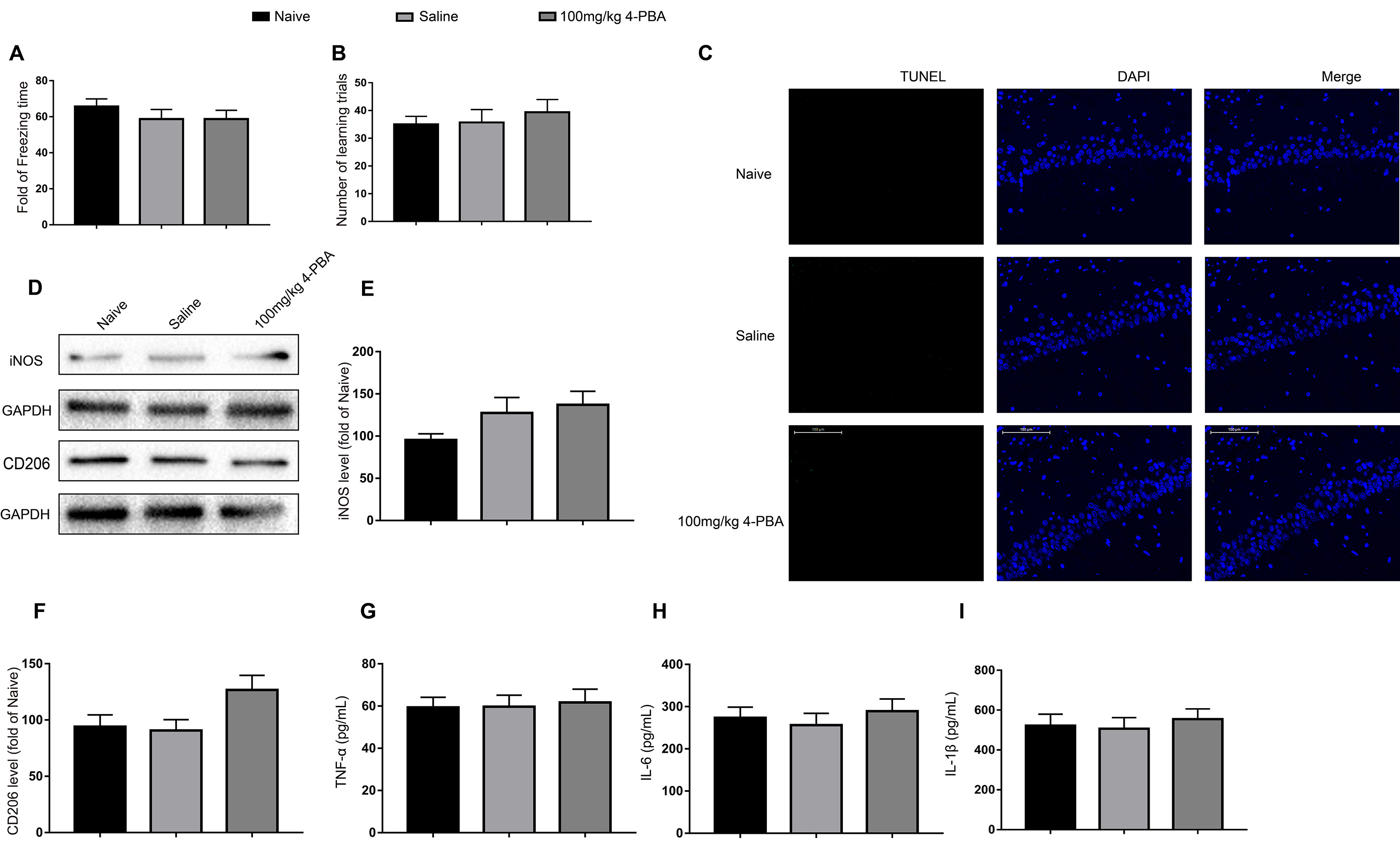
**Supplementary file 1.** 4-PBA (100mg/kg) had no neurotoxicity and proinflammatory effect in healthy rats. (A) Contextual fear response, as measured by freezing time, was determined in the rats (n=6). (B) The number of learning trials was recorded to analyze Y-maze test (n=6). (C) The TUNEL assay was performed to determine the extent of apoptosis in the CA1 area of the hippocampus. Scale bar, 100 μm. (D) The expression of iNOS and CD206 was detected by Western blotting using specific antibodies in the hippocampus of rats. Each blot is representative of three experiments. (E and F) Expression of iNOS and CD206 was quantified and normalized to GAPDH levels. Each value was then expressed relative to the naïve group, which was set to 100 (n=3). (G-I) The levels of proinflammatory factors TNF-α, IL-6, and IL-1β were detected by ELISA (n=3). Data are representative of 3 independent experiments. ^*^*P* < 0.05 vs. naive group. Data are presented as the mean ± SEM.
